# Inbreeding and homozygosity in breast cancer survival

**DOI:** 10.1038/srep16467

**Published:** 2015-11-12

**Authors:** Hauke Thomsen, Miguel Inacio da Silva Filho, Andrea Woltmann, Robert Johansson, Jorunn E. Eyfjörd, Ute Hamann, Jonas Manjer, Kerstin Enquist-Olsson, Roger Henriksson, Stefan Herms, Per Hoffmann, Bowang Chen, Stefanie Huhn, Kari Hemminki, Per Lenner, Asta Försti

**Affiliations:** 1 Division of Molecular Genetic Epidemiology, German Cancer Research Center (DKFZ), Heidelberg, Germany; 2 Department of Radiation Sciences & Oncology, Umeå University, Umeå, Sweden; 3 Cancer Research Laboratory, Faculty of Medicine, University of Iceland, Reykjavik, Iceland; 4 Molecular Genetics of Breast Cancer, German Cancer Research Center (DKFZ), Heidelberg, Germany; 5 The Malmö Diet and Cancer Study, Lund University, Malmö, Sweden; 6 Department of Plastic Surgery, Skåne University Hospital, Malmö, Lund University, Malmö, Sweden; 7 Department of Public Health and Clinical Medicine/Nutritional Research, Umeå University, Umeå, Sweden; 8 Cancer Center Stockholm Gotland, Stockholm, Sweden; 9 Institute of Human Genetics, Department of Genomics, University of Bonn, Bonn, Germany; 10 Division of Medical Genetics and Department of Biomedicine, University of Basel, Basel, Switzerland; 11 Center for Primary Health Care Research, Clinical Research Center, Lund University, Malmö, Sweden

## Abstract

Genome-wide association studies (GWASs) help to understand the effects of single nucleotide polymorphisms (SNPs) on breast cancer (BC) progression and survival. We performed multiple analyses on data from a previously conducted GWAS for the influence of individual SNPs, runs of homozygosity (ROHs) and inbreeding on BC survival. (I.) The association of individual SNPs indicated no differences in the proportions of homozygous individuals among short-time survivors (STSs) and long-time survivors (LTSs). (II.) The analysis revealed differences among the populations for the number of ROHs per person and the total and average length of ROHs per person and among LTSs and STSs for the number of ROHs per person. (III.) Common ROHs at particular genomic positions were nominally more frequent among LTSs than in STSs. Common ROHs showed significant evidence for natural selection (iHS, Tajima’s D, Fay-Wu’s H). Most regions could be linked to genes related to BC progression or treatment. (IV.) Results were supported by a higher level of inbreeding among LTSs. Our results showed that an increased level of homozygosity may result in a preference of individuals during BC treatment. Although common ROHs were short, variants within ROHs might favor survival of BC and may function in a recessive manner.

Breast cancer (BC) is the most common cancer among women, comprising about 23% of all female cancers. Each year, nearly 1.67 million new cases are diagnosed and almost 522 000 women die of this disease^1^. It has been shown that survival of BC is partly heritable due to yet unknown genetic factors^2^. Further knowledge about the effects of genetic variants on BC survival will help to predict the patient’s individual risk for disease progression and survival probabilities and to develop new and better therapies and preventive strategies. Within the last six years 34 genome-wide association studies (GWASs) on BC have been performed identifying 194 new susceptibility loci (http://www.genome.gov/gwastudies). Their identification has provided important and novel insights into the biology of BC^3^. In addition, three GWASs have been conducted on BC survival but they only led to the discovery of three prognostic loci^4^,^5^,^6^.

A more global view on the GWAS data can reveal new insights in cancer formation and progression and give new clues for further investigations. The majority of cancer predisposition genes that have been identified through GWASs function in a co-dominant manner, and the studies have not found evidence for recessively functioning disease loci. From the biological point of view it is reasonable to assume that tumors may also appear as an autosomal recessive disease. This is supported by a study that shows an increased cancer incidence associated with consanguinity and higher risk in populations characterized by a higher degree of inbreeding and corresponding homozygosity^7^. As a result, affected individuals are more often homozygous for sequence variants that underlay the disease^8^.

Unfortunately, conventional methods to analyze GWASs and whole exome or whole genome sequencing studies are prone to overlook variants which might exert a recessive effect on the risk of a disease, either as homozygotes or compound heterozygotes^9^. Therefore, a variety of studies have been performed to identify regions with runs of homozygosity (ROHs) and to prove their recessive effects on the risk of complex diseases and traits^10^,^11^,^12^,^13^,^14^,^15^. Several studies have even investigated whether ROHs are associated with an increased risk of developing cancers such as breast, colorectal, lung, prostate, and head/neck^3^,^16^,^17^,^18^. While Assie *et al.* showed increased germline homozygosity at specific loci in cancer cases, Orloff *et al.,* and Spain *et al.* reported a significantly increased frequency of homozygous regions in cases compared with controls^16^,^17^,^18^. However, Enciso-Mora *et al.* provided no strong evidence for homozygosity as a risk factor for breast or prostate cancer^3^.

We conducted a whole-genome homozygosity analysis on BC survival based on our GWAS data^19^. The aim of our study was to examine whether extended homozygosity is associated with an increased or decreased survival of BC and to search for novel recessively acting disease loci.

## Results

**Figure 1:**
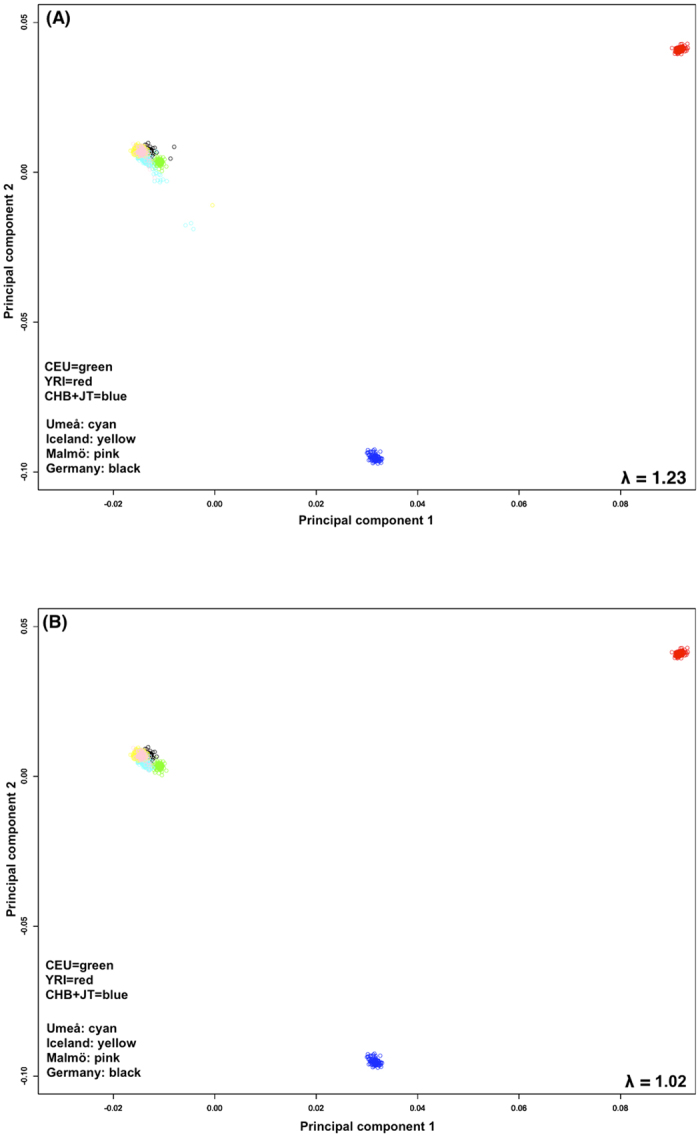
Population stratification before (**A**) and after (**B**) quality control (principal component analysis together with the HapMap individuals with CEU: green, YRI: red, CHB + JT: blue).

The GWAS data were subjected to rigorous quality control based on standard protocols^20^. The data set was then critically evaluated for ancestral differences by principal component analysis. Figure 1(A,B) show plots of the first two principal components for the study samples and the corresponding HapMap data before and after exclusion of outliers. There was a good match with the samples of European ancestry. After quality control association between homozygosity and BC survival was tested in three ways.

### Genome-wide assessment of associations between homozygosity at single SNPs and BC survival

The mean of the overall proportion of homozygosity for the complete SNP set was significantly lower in STSs as compared to LTSs (*P* = 0.05). Subsequently, a test for the genome-wide assessment of homozygosity and BC survival was performed on a SNP-by-SNP basis. The corresponding QQ-plot of the *P*-values is shown in the supplemental Figure 1. Results for the best SNPs with *P* < 1*10^−4^ are shown in Table 1. The most strongly associated SNP was rs9754606 (chr3: 192 220 488 bp; *P*_homoz_ = 2.2*10^−6^; chi^2^ = 22.41). The false discovery rate (FDR) controlled at some arbitrary level of q* did not fall below the level of q* < 0.05 to indicate globally significant association.

### Identification of individual ROHs per person and association between ROHs and BC survival

**Figure 2:**
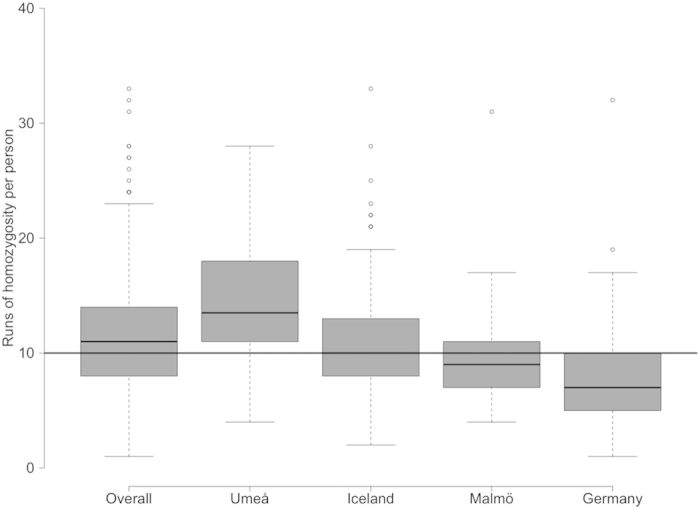
Distribution of the number of runs of homozygosity in the different population subsets.

**Figure 3:**
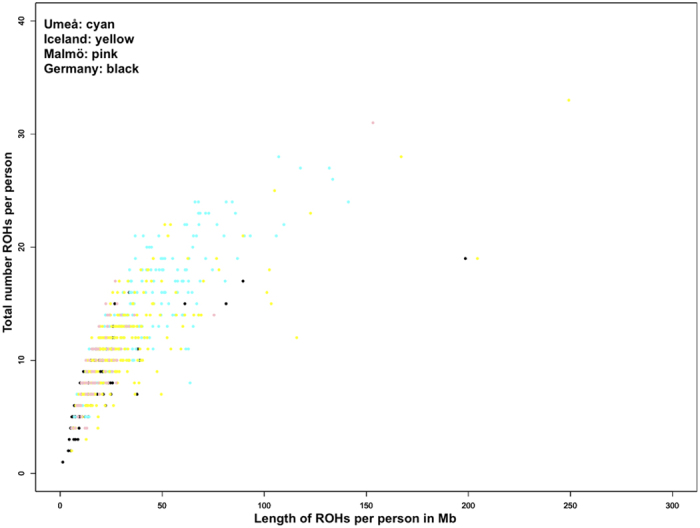
Total number and length of runs of homozygosity per person by population subsets.

Within our sample set we identified a total of 7646 individual ROHs larger than 1000 kb across all 675 individuals (3608 in the 340 STSs and 4038 in the 335 LTSs). The average length of these ROHs was 2598.59 kb. For each individual, an average of 11.32 ROH segments were detected, which covered in total 8.1% of the human genome. An overview of the distribution of ROHs in the different populations is represented in Fig. 2, showing that most of the individuals of the Umeå population had about 10 to 20 ROHs per person whereas the German population had its mean at eight ROHs per person. Figure 3 shows the individual numbers of ROHs per person in relation to the total length of the ROHs in Mb (all ROHs above 1 Mb differentiated by population). Data points for the German and Malmö subgroups were generally narrowly distributed along both axes, indicating that these individuals had few, relatively short ROHs per person. The two other sample groups were much more widely spread along both axes, reflecting the presence of many and much longer ROHs per person.

Overall, the mean ROH size per person as well as the total length of ROHs per person was not different between STSs and LTSs (Table 2). However, the number of ROHs per person was significantly higher in LTSs than in STSs (*P* = 0.0001). Even though the population identifier used as a covariate in a generalized linear model had a strong effect (*P* < 4.55*10^−6^), the difference in the number of ROHs between STSs and LTSs was still significant at *P* = 0.049. After applying a permutation test the number of ROHs per person remained significant (*P* = 0.049), but the origin of the different populations also stayed significant (*P* < 1*10^−6^), indicating the population as a confounder.

Due to the observed differences in the number of ROHs per person, the burden analysis was extended to the population subgroups (Table 2). In none of the subgroups, any of the calculated parameters differed significantly between STSs and LTSs, even though LTSs of the Icelandic subpopulation showed marginally higher numbers of ROHs per person (*P* = 0.08). Table 2 also gives an overview of the differences among the populations in general. The means of the number of ROHs per person, the total and the average length of the ROHs per person were significantly smaller in the German subset than in the other three subpopulations (*P* = 0.0003). Compared to the Umeå subset, the Malmö and Icelandic subset showed significantly smaller ROHs per person and smaller total and average ROH size (*P* = 0.003 and *P* = 0.0001, respectively).

### Common ROH regions and association with BC survival

For a more powerful association analysis between BC survival and ROHs all individuals of the different populations were pooled. A total of 2287 groups for overlapping regions of homozygosity were formed, of which 143 ROHs fulfilled the criteria for the identification of common ROHs (a consensus SNP set representing the minimal overlapping of 75 SNPs in ≥5 samples or pools being homozygous in either STSs only or LTSs only). None of the common ROH regions were associated with BC survival after correction for multiple testing. However, seven regions were associated at a suggestive level (*P* < 0.05). Another four regions with a *P*-value <0.05 were present in only four individuals, but also following the general pattern of the ROH regions being exclusively present in LTSs and absent in the STSs and thus, associated with longer survival of BC. As shown in Table 3, the LTSs with longer ROHs were mainly members of the Icelandic and Umeå subgroups, whereas among the STSs only one German woman carried ROH3 and ROH7. None of these overlapping ROHs shown in Table 3 encompassed the centromeric regions. The accompanying inspection of the data for copy number variants (CNVs) resulted in 10.800 CNVs. An average, 16 CNVs were discovered per sample. The average CNV size was 107 kb. After a detailed scan no CNVs were detected within the overlapping ROHs.

All common ROH regions were tested for differences among all STSs and LTSs of our sample with respect to the proportions of SNPs being homozygous. Table 3 shows the corresponding *P*-values of the one-tailed t-test for each ROH. Six ROHs showed highly significant differences. The right column of Table 3 shows, that for all common ROHs except for ROH6 the H_0_ could be rejected. FDR_ROH_ were significantly smaller than FDR_GWAS_, indicating that ROHs are not inferior to GWAS results. None of the SNPs on the SNP-by-SNP based test (*P* < 1*10^−4^) was overlapping with any of the common ROH regions.

### Natural selection as a cause of ROHs

ROHs have been suggested to derive from three possible mechanisms: relatedness due to demographic events (e.g. bottleneck events, founder effects or population isolation), natural selection or recent parental relatedness (inbreeding)^21^. In order to assess the influence of selection on the most promising ROH regions, three estimates were used, Tajima’s D, iHS and Fay Wu’s H^22^,^23^,^24^. Every ROH of interest showed highly significant values for all three estimates (iHS > 2.0, Tajimas’ D > 2.0, and Fay Wu’s H ≪ −10; Table 3), indicating that each of the eleven most promising ROH regions might be the result of a selective sweep.

### Inbreeding and association between homozygosity and BC survival

Next, we calculated the inbreeding coefficients for all samples using the SNP data, i.e. the relationship between haplotypes within an individual. Three estimates were used: one based on the variance of additive genetic values (F I), the second based on SNP homozygosity (F II) and the third based upon the correlation between uniting gametes (F III)^25^. The means and standard deviations (SDs) for F II in STSs and LTSs were 0.004 (SD 0.016) and 0.006 (SD 0.012), respectively, and significantly different from each other (*P* = 0.03, by t test and by regression of F II on survival as a binary trait (0/1) in a generalized linear model using glm() in R). This suggests that LTSs were in general more inbred than STSs. However, inbreeding coefficients F I and F III did not differ significantly between STSs and LTSs for the overall data set, but means and SDs for F III in STSs were still lower with 0.005 (SD 0.015) than in LTSs with 0.006 (SD 0.011), which supports the differences shown above. Breaking down the analysis of the overall genome to single chromosomes revealed, that the primary source of differences in inbreeding was due to chromosome 9 and 15, for which we detected significantly higher values for all three inbreeding coefficients in LTSs at *P* = 0.01 (data not shown).

Testing each population subgroup for any differences of the inbreeding coefficients between STSs and LTSs did not show any significant results.

**Figure 4:**
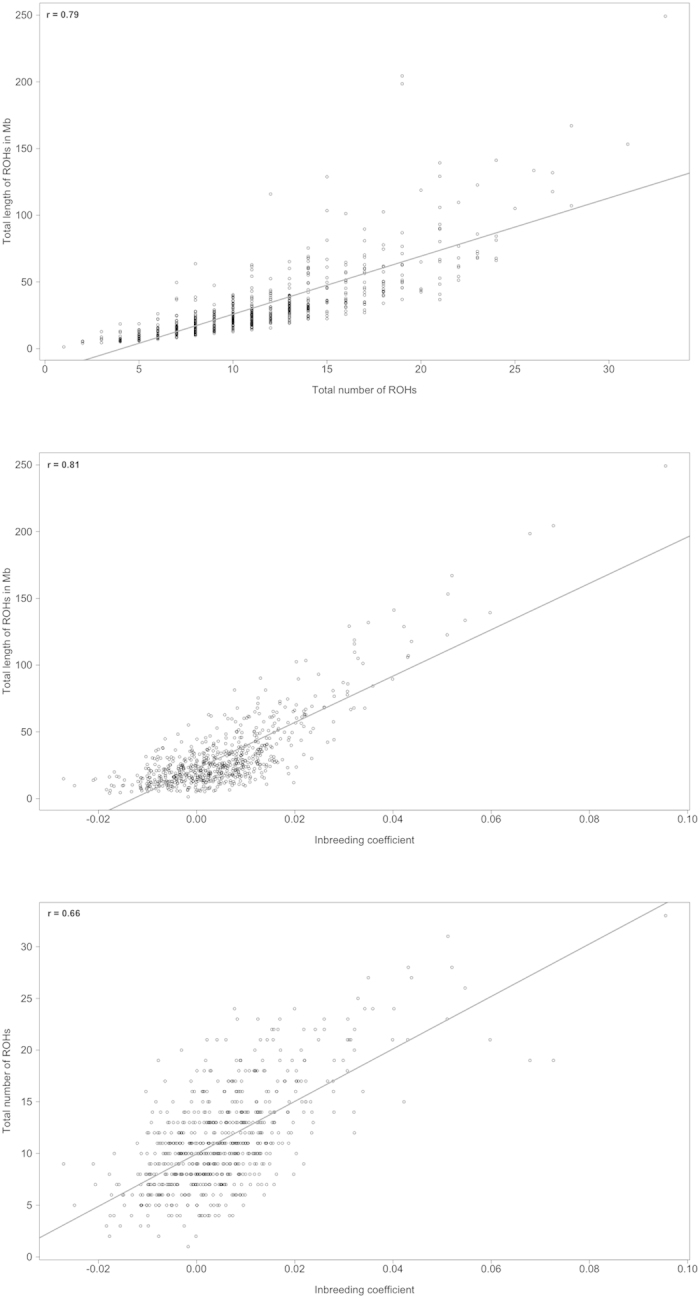
Correlations between consanguinity measures.

To illustrate the relationship between inbreeding and ROHs we assessed correlations between different consanguinity measures as shown in Fig. 4. Due to extreme values in the total number of ROHs one outlier of the German cases was excluded. The total length of individual ROHs was highly correlated with the total number of ROHs per individual (r = 0.79, *P* < 0.0001). A similar correlation was estimated between the total number of ROHs per individual and the individual inbreeding coefficient F II (r = 0.66, *P* < 0.0001). The highest correlation was detected between the total length of ROHs per individual and the individual inbreeding coefficient (r = 0.81, *P* < 0.0001). The results show that the number of ROHs and their corresponding length is associated with the level of inbreeding of each individual.

Finally, we checked for an association between homozygosity represented by the genomic inbreeding coefficient F_ROH_ and survival of BC. The overall means and SDs for F_ROH_ in STSs and LTSs were 0.0112 (SD 0.015) and 0.0128 (SD 0.010). The true difference in means was greater than zero at *P* = 0.05. For the subpopulations no significant differences were observed except for the Islandic group with a mean of 0.010 (SD 0.008) for STSs and 0.012 (SD 0.011) for LTSs at *P* = 0.07. On a chromosome-wise level inbreeding coefficients for chromosome 15 were also significantly higher in LTSs with 0.045 (SD 0.06) than for STSs with 0.029 (SD 0.03) (*P* = 0.04). For chromosome 9 the trend was similar with a mean of F_ROH_ for STSs with 0.025 (SD 0.03) and for LTSs 0.029 (SD 0.05) (*P* = 0.24).

## Discussion

To our knowledge the current work is the first analysis of the influence of genomic homozygosity on the survival of BC patients. Homozygosity can be caused by demographic events, consanguinity/inbreeding or selective pressure. In our study, most of the ROHs were relatively short excluding consanguinity as the cause of inbreeding, although inbreeding coefficients point to a certain level of relatedness. On the other hand, all of the ROHs of interest showed highly significant evidence for natural selection (iHS, Tajima’s D, Fay-Wu’s H)^23^. Thus, the influence of selective pressure on the ROH length cannot be excluded either.

We show some evidence that survival of BC may be associated with increased homozygosity and an increased level of inbreeding. Our stringent quality control prior to the analysis provided the required certainty of no bias due to population stratification for the analysis on a SNP-by-SNP basis. No significant differences in the proportion of homozygous individuals among STSs and LTSs were observed in the SNP-by-SNP analysis.

Further downstream analysis indicated significant differences among the populations in terms of the number of ROHs per person and the total and average length of ROHs per person. These differences are well known and have been used as a resource for studying human genetic diversity and evolutionary history^21^. The origin of the different populations had a significant impact on the differences of the number of ROHs per person and the total and average length of ROHs per person. However, the difference in the number of ROHs per person between STSs and LTSs remained significant (*P* = 0.049) by using a generalized linear model with population identifier as a covariate, and it was confirmed by a permutation test.

As a consequence of the significant differences the total number, the total length of ROHs and the mean ROHs sizes per person were analyzed separately for each subpopulation. Although the overall analysis showed an increased number of ROHs among LTSs, the stratified analysis did not show any significant differences. A possible reason might be the relatively small number of individuals per subgroup. However, the patterns followed the same trend in the Icelandic and Malmö subgroup.

Most importantly, several of the ROHs were significantly more homozygous among LTSs than among STSs, and the FDR was also significantly lower. Some of the common ROHs identified in our analysis also overlap with long contiguous stretches of homozygosity from another study but are not due to chromosomal abnormalities or common copy number variants^26^. Intriguingly, several regions identified as suggestive ROHs harbor genes that are associated with progression and metastasis in BC, such as the *GPATCH2* gene on 1q41^27^. This region (ROH3, Table 3) was homozygous in eight LTSs but only in one STS.

Another important region with influence on BC survival was identified on chromosome 15 (ROH2). Within this region the *GRINL1A* complex transcription unit (CTU) represents a naturally occurring read through transcription between the neighboring genes *MYZAP* (*GCOM1*) and *POLR2M* (*GRINL1A*)^28^. Interestingly, *GCOM1* has been identified as an estrogen receptor β (*ERβ*) target gene^29^.

The second homozygous region on chromosome 15 (ROH4) hosts two genes of the gamma-aminobutyric acid A receptor family (*GABRB3* and *GABRB5*), that are related to the chemokinesis and chemotaxis in MDA-MB-468 human breast carcinoma cells^30^.

For several other homozygous regions such as ROH5, ROH6, ROH7, ROH9 and ROH10 genes have been identified with an association for BC or BC progression. These genes may modify disease risk or tumor progression, or they may work as markers of protection, transcription co-activators, or oxidative stress-modifying genes^31^,^32^,^33^,^34^,^35^,^36^.

One of the most striking results of our investigation was the higher degree of homozygosity among LTSs of BC, which is represented by an increased measure of the inbreeding coefficient. These results are in good agreement with the detection of more LTSs within individuals of higher number of ROHs or increased length of homozygous stretches. Further analysis of common ROHs did not result in genome-wide significant differences in survival, but all the regions reflected the same pattern of showing more or solely LTSs being homozygous for specific regions. Most of these regions could even be linked to genes related to progression or treatment in BC. Thus, there seems to be evidence for an association between homozygosity and survival of BC.

The remaining question is whether increased homozygosity in certain regions of the genome supports longer survival of BC or in a reverse way whether increased homozygosity has originated from the fact that patients being homozygous for certain loci respond better to treatment and therefore have better survival.

A possible explanation for the results of increased homozygosity among LTSs may be a relative preference of regions carrying no mutation at all compared with those that carry deleterious mutations in a homozygous or heterozygous status. As such, the regions of homozygosity may reflect a certain degree of genomic resistance against the challenges of chemotherapeutic treatment as compared with heterozygous genotypes. A great example for a similar pattern is provided by the *CHEK2* locus, where the CHEK2*1100delC heterozygosity was associated with a 1.4-fold risk of early death in BC patients compared to noncarriers^37^. It is one of the most recent and well-documented examples for a genetic factor influencing long-term prognosis of women with BC. An earlier publication also showed that heterozygote carriers of the *NBN* founder mutation are under higher risk to develop BC and die earlier^38^. Overall, there seems to be some variation of genotypes within patients that will help them to survive the applied treatment better than others. Such genotypes, either alone, in interaction with each other or in combination with specific drugs or treatments may result in better treatment outcome, decreased side effects or improved survival. Therefore, the discovery and understanding of such genotypes may be vital for the improvement of cancer therapy.

## Material and Methods

The GWAS on BC survival was a population based case-only study, in which the BC patients were divided in two groups based on their survival time^19^. A group of 369 women with short-time survival (STS, less than 6 years after BC diagnosis) was compared with a group of 369 women with long-time survival (LTS, ≥11 years after BC diagnosis). The cases with STS and LTS were selected from four cohorts and matched for age (<40, 40–49, 50–59 and ≥60 years), period of diagnosis (1985–1989, 1990–1994 and 1995-) and the corresponding cohort: 1) 96 STSs and 96 LTSs from the Västerbotten intervention project, the mammary screening project and from the Department of Oncology, Norrlands University Hospital, Umeå, Sweden^39^; 2) 44 STSs and 44 LTSs from Malmö Diet and Cancer Study, Malmö, Sweden^40^; 3) 82 STSs and 14 LTSs from the Städtisches Klinikum Karlsruhe and Deutsches Krebsforschungszentrum Breast Cancer Study (SKKDKFZS) consisting of women between 21–93 years of age at diagnosis with pathologically confirmed BC recruited at the Städtisches Klinikum Karlruhe, Karlsruhe, Germany from 1993–2005^41^; and another 68 LTSs from the Umeå cohort; 4) 147 STSs and 147 LTSs from the Icelandic Cancer Society and University of Iceland Biobank^42^. The STSs and LTSs were identified from the cohorts by record linkage to the regional cancer registries. Follow-up was performed until 2008 and the data were available for every patient. Disease stage of the patients was categorized from 0 to IV. STSs tended to have tumors of higher stage than LTSs^19^.

### Ethics statement

The studies were coordinated at the German Cancer Research Center (DKFZ) with samples and information obtained with full informed consent and national ethical review board approval [Dnr 07-14IM] in accordance with the Declaration of Helsinki.

### Genotyping and quality control

For all samples ~300 000 tagging single nucleotide polymorphisms (SNPs) were genotyped using the Illumina HumanCytoSNP-12v1. Quality control procedures were based on standard protocols using PLINK software (v1.07) and R, v3.0.2 (R Foundation for Statistical Computing, Vienna, Austria)^19^,^20^,^43^.

To exclude individuals with non-Western European ancestry, data of the STSs and LTSs were merged with data obtained from the International HapMap Project^44^. Principal component analysis was used to identify population outliers. The remaining individuals matched genetically well to the HapMap samples with northern and western European ancestry (CEU). After stringent quality control the final data set consisted of 340 STRs and 335 LTRs with genotyping information for 232 478 autosomal SNPs.

### Genome-wide assessment of homozygosity at individual SNPs and BC survival

Motivated by the observation of high frequencies of germline homozygosity at specific markers in cancer cases by Assie *et al.* an initial test as described by Spain *et al.* was performed for any association between homozygosity (whether for the major or minor allele) and BC survival on a SNP-by-SNP basis in our entire sample series based on a chi^2^-test with the number of homozygotes and heterozygotes at each SNP in STSs and LTSs^16^,^17^. To control the problem of multiple testing the false discovery rate (FDR) was calculated and controlled at an arbitrary level q*^45^.

### Identification of runs of homozygosity

We defined ROHs following recommendations in Howrigan *et al.*^46^. ROHs were detected using PLINK (v1.07) software. The ROH tool moves a sliding window of SNPs across the entire individual genome. To prevent for any genotyping errors or other sources of artificial heterozygosity, such as paralogous sequences within a stretch of truly homozygous SNPs and, hence, to balance the number and size of ROHs, no heterozygous SNPs were permitted in any window. We set the remaining options to default values (including at most three missing calls per window, thereby ensuring >90% positive-predictive value of each ROH), except that we varied the parameters for “homozyg-snp” option according to our heuristic preferences for defining ROHs as detailed below. Subsequent statistical analyses were performed using packages available in R (version 3.0.2; R Foundation for Statistical Computing, Vienna, Austria). Comparison of the distribution of categorical variables was performed using the chi^2^-test with *P*-values based on Monte Carlo simulations as implemented in the R statistics package. To compare the difference in the average number of ROHs between STSs and LTSs, we used the Student t-test. To account for any confounding due to the different population background of the samples a generalized linear model was applied with the population identifier as a covariate. A permutation test based on the permutation of the regressor residuals in the R package “glmperm” was used to secure the results^47^,^48^.

### Criteria for the detection of runs of homozygosity

The initial search for ROHs along each individual’s genome was performed using PLINK with a specified length of 75 consecutive SNPs. The reason for choosing 75 SNPs is based on the likelihood of observing 75 consecutive chance events that can be calculated as follows^14^: in our BC data mean heterozygosity was calculated to be around 35%. Thus, given 232 478 SNPs and 675 individuals, a minimum length of 51 SNPs would be required to produce <5% randomly generated ROHs across all subjects ((1–0.35)^51^ × 232 478 × 675 = 0.04; ~4%). A consequence of linkage disequilibrium (LD) is that SNP genotypes are not always independent, thereby inflating the probability of chance occurrences of biologically meaningless ROHs. Analyses were based on the pairwise LD SNP pruning function of PLINK with a default value of r^2^ > 0.8, that is necessary to declare that one SNP tags another. Restricting the search of tags to within 250 kb showed 164 484 separable tag groups, representing a 30% reduction of information compared with the original number of SNPs. Thus, ROHs of length 75 were used to approximate the degrees of freedom of 51 independent SNP calls.

In the next step PLINK software and packages in R were used to identify a list of ‘common’ ROHs with a minimum of 75 consecutive SNPs for at least two individuals and with each ROH having identical start and end location across the individuals in whom that ROH was observed. The “homozyg-group” option of the PLINK package produced a file of the ROH regions separated into pools containing the number of STSs and LTSs carrying the same ROH. Corresponding information of the PLINK output file was used in assisting with the interpretation of the results. We defined that pools with more than five individuals and at least 75 identical SNPs being homozygous among the individuals in the same genomic region are treated as common ROHs. In addition, pools being homozygous in either STSs only or LTSs only were included to the list of common ROHs. Copy number variants were detected for each individual using R with no restriction towards the number of SNPs or the length of the CNVs and compared with common ROHs.

An additional test was looking for differences of the average proportion of homozygous genotypes between STSs and LTSs. For common ROH regions the proportion of homozygous genotypes was calculated for all STSs and LTSs separately, and the significance of the difference was tested by a one-tailed t-test. Likewise, for each common ROH region p-values of the above stated SNP-by-SNP test were also compared with those obtained from the prior standard single-SNP GWAS. According to the concept of non-inferiority trials, the false discovery rate (FDR) was computed for both sets of p-values and tested for equivalence by a paired t test^49^. The null hypothesis states that the FDR of the ROHs will be equal to the FDR from GWAS for the same region:


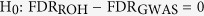


As an alternative hypothesis the FDR was smaller for ROH:


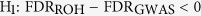


This would imply, that ROH are superior to GWAS.

### Testing of natural selection as a cause of ROHs

For common ROH regions we used three metrics to investigate the selective pressure on each of the ROH. The integrated haplotype score (iHS) is based on linkage disequilibrium (LD) surrounding a positively selected allele compared with background, providing evidence of recent positive selection at a locus^23^. A iHS score ≥2.0 reflects the fact that haplotypes on the ancestral background are longer compared with those on the derived allelic background. Episodes of selection tend to skew SNP frequencies in different directions. We estimated values for Tajima’s D and Fay and Wu’s H based on the frequencies of SNPs segregating in the region of interest^50^,^51^. iHS, Tajima’s D, and Fay and Wu’s H metrics were obtained from Haplotter Software (University of Chicago, Chicago, IL, USA; http://haplotter.uchicago.edu/selection/)^23^.

### Testing the effect of inbreeding on survival

To test whether inbreeding influenced the survival of BC patients, the three inbreeding measures F I, F II and F III using the package Genome-wide Complex Trait Analysis (GCTA) were estimated for each individual, and then tested for correlation with survival of BC^25^. As the covariate age at diagnosis did not show significant influence in prior tests, it was omitted from the analysis. Besides that, a genomic measure of individual homozygosity (F_ROH_) was calculated as proposed by McQuillan *et al.*^52^, in which L_ROH_ is the sum of ROH per individual above a certain criterion length (i.e. 1000 kb as in the publication) and L_AUTO_ is the total SNP-mappable autosomal genome length (2.67 × 10^9^ bp): F_ROH_ = ∑ L_ROH_/L_AUTO_. For this calculation centromeres were excluded, because they are characterized as long genomic stretches devoid of SNPs and tend to inflate estimates of autozygosity^52^.

## Additional Information

**How to cite this article**: Thomsen, H. *et al.* Inbreeding and homozygosity in breast cancer survival. *Sci. Rep.* **5**, 16467; doi: 10.1038/srep16467 (2015).

## Supplementary Material

Supplementary Information

## Figures and Tables

**Table 1:** Association between homozygosity and time of survival for individual SNPs.

SNP	CHR	BP	STSs homozygotes	STSs heterozygotes	LTSs homozygotes	LTSs heterozygotes	chi^2^	P*	q*
rs9754606	3	192220488	201	139	137	198	22.41	2.20-06	0.30
rs6026932	20	57527300	142	197	201	134	22.11	2.56-06	0.30
rs16972118	13	107644377	265	75	210	125	18.83	1.42-05	0.44
rs812046	3	123899115	254	86	294	41	18.82	1.43-05	0.44
rs12875580	13	36924447	250	90	290	44	18.70	1.52-05	0.44
rs1908968	4	143488152	229	111	171	164	18.58	1.62-05	0.44
rs6465946	7	103375388	141	199	194	141	18.24	1.94-05	0.44
rs2282086	9	126064894	262	78	208	127	17.88	2.35-05	0.44
rs7923443	10	54227574	289	49	242	93	17.78	2.47-05	0.44
rs10102621	8	10178285	216	122	160	175	17.78	2.47-05	0.44
rs4957981	5	111169455	190	149	240	95	17.74	2.53-05	0.44
rs6100474	20	57525991	151	189	203	132	17.72	2.55-05	0.44
rs2322122	4	166675289	159	181	210	124	17.64	2.65-05	0.44
rs6438763	3	123945816	256	84	294	41	17.38	3.05-05	0.44
rs9855669	3	123993453	256	84	294	41	17.38	3.05-05	0.44
rs4425430	4	66023562	270	70	304	31	17.03	3.66-05	0.48
rs11647013	16	87674024	193	147	241	94	16.92	3.88-05	0.53
rs7518793	1	9634074	208	132	254	81	16.75	4.25-05	0.54
rs1711656	10	131131239	242	98	188	147	16.54	4.75-05	0.55
rs4937909	11	133947031	268	72	302	33	16.47	4.92-05	0.56
rs1939008	11	102161633	227	113	173	162	15.98	6.38-05	0.67
rs6482736	10	131115541	243	97	190	145	15.97	6.42-05	0.67
rs10044914	5	107348748	225	115	171	164	15.93	6.56-05	0.67
rs627206	4	16759042	280	60	232	103	15.80	7.01-05	0.67
rs9900188	17	10102644	293	47	248	87	15.64	7.63-05	0.69
rs2829045	21	24701003	230	110	271	64	15.48	8.33-05	0.71
rs11234894	11	86357222	162	178	210	125	15.42	8.57-05	0.71
rs1559631	2	53269759	263	77	213	122	15.39	8.73-05	0.71
rs17589261	8	136263976	232	107	180	155	15.33	9.00-05	0.71
rs2253812	10	119669249	245	95	283	52	15.27	9.28-05	0.71
rs17222478	9	126105657	275	65	227	108	15.24	9.46-05	0.71

^*^P was calculated using a simple 2*2 chi^2^-test based on the number of homozygotes and heterozygotes at each SNP in short-time survivors (STRs) and long-time-survivors (LTRs).

^*^q values representing the false discovery rate (FDR).

**Table 2:** Burden analysis of ROH for the entire data set and each subset.

Entire data set	STS^a^ (n = 340)	LTS^b^ (n = 335)	*P*
Total number of ROHs	3608	4038	
Number of ROHs per person	10.61	12.05	**0.0001**
Total length of ROHs per person, kb	30205	34466	0.11
Mean ROH size per person, kb	2545	2652	0.29
**Umeå set**	**STS^a^ (n = 77)**	**LTS^b^ (n = 137)**	***P***
Total number of ROHs	1099	1916	
Number of ROHs per person	14.27	13.98	0.68
Total length of ROHs per person, kb	41820	41714	0.97
Mean ROH size per person, kb	2829	2794	0.81
**Iceland subset**	**STS^a^ (n = 141)**	**LTS^b^ (n = 143)**	***P***
Total number of ROHs	1477	1618	
Number of ROHs per person	10.47	11.31	0.08
Total length of ROHs per person, kb	28594	33164	0.15
Mean ROH size per person, kb	2580	2744	0.28
**Malmö subset**	**STS^a^ (n = 43)**	**LTS^b^ (n = 41)**	***P***
Total number of ROHs	380	406	
Number of ROHs per person	8.83	9.90	0.19
Total length of ROHs per person, kb	19351	20467	0.76
Mean ROH size per person, kb	2027	2031	0.97
**German subset**	**STS^a^ (n = 79)**	**LTS^b^ (n = 14)**	***P***
Total number of ROHs	652	98	
Number of ROHs per person	8.25	7.00	0.85
Total length of ROHs per person, kb	27666	17836	0.32
Mean ROH size per person, kb	2485	2149	0.41

^a^Short-time survivors.

^b^Long-time survivors.

**Table 3:** List of ROHs associated with BC survival.

ROH	Chr.	Start (bp)	End (bp)	STS^a^,^b^	LTS^a^,^b^	Chi^2^	*P*^c^	*P*_hom_^d^	*P*_diff_^e^	iHS _max._^f^	Tajima D_max_^g^	Fay Wu’s H_min_.^h^	Genes						
ROH1	2	40485599	41849839	0	6 (3 I, 3 U)	6.14	**0.01**	0.11	**<0.001**	4.24	3.27	−86.69	*SLC8A1*						
ROH2	15	55462030	56123292	0	6 (1 I, 5 U)	6.14	**0.01**	**<0.001**	**<0.001**	2.49	3.23	−44.05	*GRINL1A, GCOM1*						
ROH3	1	215442447	216299477	1 (G)	8 (4 I, 3 U, 1 M)	5.62	**0.01**	0.15	**<0.001**	3.42	1.99	−39.03	*UBBP2, GPATCH2, SPATA17*						
ROH4	15	24085492	24771914	0	5 (2 I, 3 U)	5.11	**0.02**	0.17	**0.002**	2.61	2.58	−48.22	*GABRA5, GABRB3*						
ROH5	5	52523352	53220309	1 (I)	7 (4 I, 3 U)	4.64	**0.03**	**<0.001**	**<0.001**	2.65	2.50	−29.87	*ASSP9, NDUFS4, FST*						
ROH6	10	80900995	82333991	1 (U)	7 (2 I, 5 U)	4.64	**0.03**	**0.008**	<0.81	2.86	2.85	−33.09	*ANXA11, EIF5AP1, MAT1A, SFTPA1B, SFTPA2B, SFTPD, MBL1P1, C10orf57, TSPAN14, C10orf58, DYDC2, DYDC1, EIF5AL1, PLAC9, FAM22E, TPRX1P1, TPRX1P2, EIF5AL3, SFTPA1, FAM22C, SFTPA2 FAM22B*						
ROH7	11	122870127	124132689	1(G)	7 (3 I, 4 U)	4.64	**0.03**	**0.003**	**<0.001**	2.70	3.07	−65.22	*VWA5A, NRGN, ZNF202, OR8C1P, OR8B1P, VSIG2, OR8G2, OR8B8, OR8G1, OR10D3P, OR10D1P, OR8B7P, OR8B6P, OR8B5P, OR8B2, SPA17, SIAE, SCN3B, GRAMD1B, OR8B9P, OR6M3P, OR10D5P, OR10G6, OR10G5P, OR6M2P, OR8Q1P, TBRG1, PANX3, OR8B12, OR8G5, OR10G8, OR10G9, OR10S1, OR6T1, OR4D5, OR8G7P, OR8D1, OR8D2, OR8B4, PMP22CD, OR8D4, OR8G3P, OR6 × 1, OR6M1, OR10G4, OR10G7, OR10D4P, OR10N1P, OR8F1P, OR8B3, OR8A2P, OR8B10P, OR8A1, OR8A3P, OR8 × 1P, SF3A3P2*						
ROH8	18	63092756	64110327	0	4 (2 I, 2 U)	4.08	**0.04**	0.89	**<0.001**	3.50	3.57	−64.76	*DSEL*						
ROH9	4	23473510	24501859	0	4 (2 I, 2 U)	4.08	**0.04**	0.22	**<0.001**	3.14	3.18	−65.63	*DHX15, SOD3, ATP5LP3*						
ROH10	3	150003443	151430969	0	4 (4 I)	4.08	**0.04**	**0.003**	**<0.001**	2.56	2.94	−36.76	*CP, CPA3, CPB1, FKBP1P4, GYG1, TM4SF1, PFN2, HLTF, TM4SF4, RNF13, WWTR1, COMMD2, HPS3, TM4SF18, UBQLN4P, C3orf16, FLJ40759, TMEM183B*						
ROH11	2	43910360	44989389	0	4 (2 I, 2 U)	4.08	**0.04**	**<0.001**	**<0.001**	2.61	2.86	−49.94	*PPM1B, SLC3A1, PREPL, LRPPRC, ABCG8, C2orf34*						

Chromosomal positions derived from the National Center for Biotechnology Information (NCBI), build 36, hg18.

^a^Short-time survivors (STSs), long-time survivors (LTSs), coding of the population subgroups (G = Germany, I = Iceland, M = Malmö, U = Umeå).

^b^Only one patient among STSs (G of ROH3 and ROH7) was diagnosed for Stage 3 while all other patients were evenly distributed among Stage 1 or Stage 2.

^c^Suggestive significance; based on Monte Carlo simulation as implemented in R statistics package; confirmed with Fisher’s exact test.

^d^P values for H_0_ : μ_STSs_ = μ_LTSs_; H_1_ : μ_STSs_ < μ_LTSs_.

^e^P values for FDR_ROH_ - FDR_GWAS_.

^d^Represents maximal absolute values for iHS, derived for CEU population ancestry from Haplotter, Phase II (http://hgwen.uchicago.edu/selection/haplotter.htm).

^e^Represents maximal values for Tajima’s D, derived for European descendants from UCSC http://genome.ucsc.edu.

^f^Represents minimum values for Fay Wu’s H, derived for CEU population ancestry from Haplotter, Phase II (http://hgwen.uchicago.edu/selection/haplotter.htm).
