# Supplementary material for: Inbreeding and homozygosity in breast cancer survival
Source: Sci Rep. 2015 Nov 12;5:16467. doi: 10.1038/srep16467 (PMC4642301; doi:10.1038/srep16467)
Supplement: Supplementary Information [file srep16467-s1.pdf]

Inbreeding and homozygosity in breast cancer survival

Hauke Thomsen,[1]* Miguel Inacio da Silva Filho,[1] Andrea Woltmann,[1] Robert Johansson,[2] Jorunn E. Eyfjörd,[3] Ute Hamann,[4] Jonas Manjer,[5,6] Kerstin Enquist-Olsson,[7] Roger Henriksson,[2,8] Stefan Herms,[9,10] Per Hoffmann,[9,10] Bowang Chen,[1] Stefanie Huhn,[1] Kari Hemminki,[1,11] Per Lenner,[2] Asta Försti[1,11]

[1] Division of Molecular Genetic Epidemiology, German Cancer Research Center (DKFZ), Heidelberg, Germany

[2] Department of Radiation Sciences & Oncology, Umeå University, Umeå, Sweden

[3] Cancer Research Laboratory, Faculty of Medicine, University of Iceland, Reykjavik, Iceland

[4] Molecular Genetics of Breast Cancer, German Cancer Research Center (DKFZ), Heidelberg, Germany

[5] The Malmö Diet and Cancer Study, Lund University, Malmö, Sweden

[6] Department of Plastic Surgery, Skåne University Hospital, Malmö, Lund University, Malmö, Sweden

[7] Department of Public Health and Clinical Medicine/Nutritional Research, Umeå University, Umeå, Sweden

[8] Cancer Center Stockholm Gotland, Stockholm, Sweden

[9] Institute of Human Genetics, Department of Genomics, University of Bonn, Bonn, Germany

[10] Division of Medical Genetics and Department of Biomedicine, University of Basel, Basel, Switzerland

[11] Center for Primary Health Care Research, Clinical Research Center, Lund University, Malmö, 20502, Sweden

* Corresponding author: Dr. Hauke Thomsen

German Cancer Research Center (DKFZ)
Molecular Genetic Epidemiology C050
Im Neuenheimer Feld 580
DE-69120 Heidelberg
Tel.: +49-6221-42-1792
E-Mail: h.thomsen@dkfz-heidelberg.de

Running title: Homozygosity in breast cancer survival

The authors declare no conflict of interest.

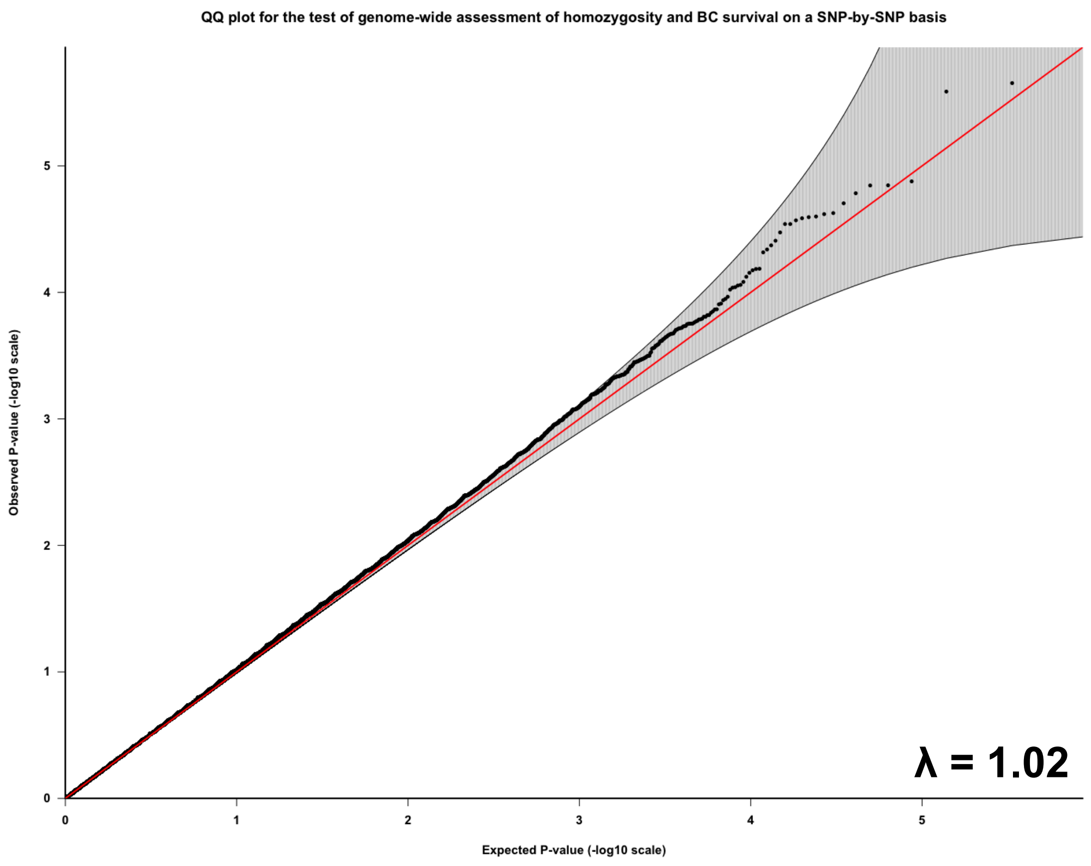

Supplemental Figure 1: QQ plot for the test of genome-wide assessment of homozygosity and BC survival on a SNP-by-SNP basis with a lambda (λ) = 1.02
